# Transcriptome Sequencing Reveals Survival Strategies and Pathogenic Potential of *Vibrio parahaemolyticus* Under Gastric Acid Stress

**DOI:** 10.3390/biology14040396

**Published:** 2025-04-10

**Authors:** Shiying Ji, Jinlin Jiang, Zhiyong Song, Yu Zhou, Lu Chen, Shiying Tang, Yingjie Pan, Yong Zhao, Haiquan Liu

**Affiliations:** 1 College of Food Science and Technology, Shanghai Ocean University, Shanghai 201306, China; 19861652095@163.com (S.J.); song1zy@163.com (Z.S.); 15224773369@163.com (Y.Z.); d220300077@st.shou.edu.cn (L.C.); tangshiying1996@163.com (S.T.); yjpan@shou.edu.cn (Y.P.); 2 Changsha Customs Technology Center, Changsha 410007, China; jiangjl2010@163.com; 3 Shanghai Engineering Research Center of Aquatic-Product Processing & Preservation, Shanghai 201306, China; 4 Laboratory of Quality & Safety Risk Assessment for Aquatic Product on Storage and Preservation (Shanghai), Ministry of Agriculture and Rural Affairs, Shanghai 201306, China; 5 Engineering Research Center of Food Thermal-Processing Technology, Shanghai Ocean University, Shanghai 201306, China; 6 Food Industry Chain Ecological Recycling Research Institute, Food Science and Technology College, Shanghai Ocean University, Shanghai 201306, China

**Keywords:** *Vibrio parahaemolyticus*, gastric acid stress, resuscitation mechanisms, pathogenicity, transcriptome sequencing

## Abstract

As a common food-borne pathogen, *Vibrio parahaemolyticus* comes into direct or indirect contact with gastric acid after ingestion. However, the mechanisms by which *Vibrio parahaemolyticus* passes through the gastric acid barrier, recovers, and causes pathogenicity remain unclear. In this study, static in vitro digestion simulation experiments showed that some strains can pass through the gastric acid barrier by utilizing microacid tolerance mechanisms and altering their survival state. Food digestion simulation experiments showed that food matrices could help bacteria escape gastric acid stress, with significantly different survival rates observed for bacteria in various food matrices after exposure to gastric acid. Interestingly, surviving *Vibrio parahaemolyticus* showed a significantly shorter growth lag time (LT) during recovery. Transcriptome sequencing (RNA-seq) analyses indicated that the bacteria adapted to gastric acid stress by regulating the two-component system through stress proteins secreted via the ribosomal pathway. Pathogenic *Vibrio parahaemolyticus* that successfully passes through the gastric acid barrier potentially exhibits enhanced pathogenicity during recovery due to the significant upregulation of virulence genes such as *tdh* and *ysc*F. This study provides a scientific basis for revealing the tolerance mechanisms of food-borne pathogens represented by *Vibrio parahaemolyticus* in the human body.

## Simple Summary

Consuming food contaminated with *Vibrio parahaemolyticus* may cause symptoms such as diarrhea. This study will investigate how *Vibrio parahaemolyticus* passes through the gastrointestinal stress environment and causes disease. It was found that some strains died after gastric acid stress, while others could be resuscitated in the intestinal fluid, although their growth, motility, and adherence were adversely affected. During the recovery process, the number of strains increased, and their morphology was restored. Additionally, the resuscitated pathogenic strains were more likely to cause diarrhea due to the upregulation of the expression of associated virulence genes. It was also found that *Vibrio parahaemolyticus* could grow on the surface of microacidic food despite it being acidophobic, so it is necessary to strengthen the detection of microacidic food that may be contaminated by *Vibrio parahaemolyticus*. Finally, we analyzed the survival strategy and pathogenic potential of *Vibrio parahaemolyticus* during gastrointestinal digestion at the genetic level, which provides a useful solution for investigating the pathogenesis of food-borne pathogens.

## 1. Introduction

Food-borne illnesses caused by bacterial contamination pose a serious threat to global public health, with food-borne and water-borne diarrheal diseases causing an estimated 2.2 million deaths annually, the majority of which are children [1]. *Vibrio parahaemolyticus* (*V. parahaemolyticus*) as a food-borne pathogen can colonize the human intestine and cause poisoning [2]. In 2015, a large-scale poisoning incident occurred in Canada that was related to the consumption of oysters contaminated by *V. parahaemolyticus* [3]. During the process of host invasion, food-borne pathogens must pass through the gastric acid barrier and reach the small intestine to cause disease [4]. While gastric acid could kill most of the bacteria that enter the stomach with food [5], *V. parahaemolyticus*, an acidophobic bacterium, has limited research on how it escapes gastric acid and colonizes in the intestine.

As acid-resistant bacteria, *Escherichia coli* strains such as EPEC employ acid-resistance strategies, such as arginine-, lysine-, and ornithine-dependent mechanisms, to pass through the gastric acid barrier and initiate severe food-borne illness within the host’s intestines [6]. A previous study has found that mild acid (hydrochloric acid) treatment enhances the acid tolerance and pathogenicity of *V. parahaemolyticus*, alters its protein expression patterns, and provides cross-protection in low-salt and thermal inactivation environments [7]. A static in vitro model was used to explore the survival rate of *V. parahaemolyticus* under different pH levels of gastric acids [8]. When the pH of the gastric acids increased to 4.0, *V. parahaemolyticus* was more likely to survive.

At present, most studies on the virulence of *V. parahaemolyticus* only focus on the effect of bile salt as a single component, but the recovery and virulence expression of bacteria do not exclusively only rely on bile salt [9]. The pathogenic site of *V. parahaemolyticus* is located in the small intestine, and bile salt, trypsin, Ca^2+^, and other components contained in intestinal fluid collectively act on bacteria together [10]. Therefore, in this research, intestinal fluid was prepared to more realistically simulate the recovery environment of strains, allowing for a deeper exploration of the virulence expression mechanism of *V. parahaemolyticus*. Additionally, transcriptome sequencing was used to reveal the underlying molecular mechanisms. This study will provide new insights into the regulation of pathogenesis in food-borne pathogens, with *V. parahaemolyticus* serving as a representative example.

## 2. Materials and Methods

### 2.1. The Selected Experimental Strains and Culture Methods

*V. parahaemolyticus* VPE5 (*tlh* positive, *tdh* and *trh* negative), VPE7 (*tlh* and *tdh* positive, *trh* negative), VPE27 (*tlh* positive, *tdh* and *trh* negative), and VPE28 (*tlh* and *tdh* positive, *trh* negative) environmental strains were used in this study [11]. *V. parahaemolyticus* frozen at −80 °C was inoculated onto Thiosulfate–Citrate–Bile salt–Sucrose (TCBS) agar and cultured at 37 °C for 9 to 14 h. Single colonies were then selected from the TCBS plate, inoculated into 15 mL trypticase soy broth (TSB) medium for activation, and cultured at 37 °C until the OD_600_ value was between 0.5 and 0.6, ensuring that the bacterial strains used in all experiments were in the logarithmic growth phase.

### 2.2. Preparation of Simulated Gastric Fluid (SGF) and Simulated Intestinal Fluid (SIF)

The preparation of SGF and SIF was based on the literature [12], with slight modifications. Solutions of KCl, KH_2_PO_4_, NaHCO_3_, NaCl, (NH_4_)_2_CO_3_, and HCl were prepared at molar concentrations (M) of 0.5, 0.5, 1, 2, 0.5, and 5.5, respectively. The 0.15 M MgCl_2_ and 0.3 M CaCl_2_ solutions were prepared by dissolving MgCl_2_·6H_2_O and CaCl_2_·2H_2_O in water, respectively. SGF was prepared by adding 6.9 mL of KCl solution, 0.9 mL of KH_2_PO_4_ solution, 12.5 mL of NaHCO_3_ solution, 11.8 mL of NaCl solution, 0.4 mL of MgCl_2_ solution, 0.5 mL of (NH_4_)_2_CO_3_ solution, 0.005 mL of CaCl_2_ solution, and 1.0 mL of HCl solution. Then, 0.8 g of pepsin (2000 U/mL) was added to the mixture. Finally, the total volume was adjusted to 500 mL with deionized water. SIF was prepared by adding 6.8 mL of KCl solution, 0.8 mL of KH_2_PO_4_ solution, 42.5 mL of NaHCO_3_ solution, 9.6 mL of NaCl solution, 1.1 mL of MgCl_2_ solution, 0.04 mL of CaCl_2_ solution, and 0.7 mL of HCl solution. Then, 0.8 g of trypsin (100 U/mL) and 4.8 g of bile (10 mM) were added to the mixture. Finally, the total volume was adjusted to 500 mL with deionized water. SGF and SIF were stored at −20 °C for later use.

### 2.3. Determination of the Experimental Gastric Acid Value

Gastric acids with pH values of 2.5, 3.5, and 4.5 were prepared according to Section 2.2. The four strains were activated according to Section 2.1 and adjusted to 10^6^–10^7^ CFU/mL. The above diluted solution was mixed 1:1 by volume with gastric acid at different pH levels and then placed in a shaker (110 rpm, 37 °C) for 2 h. Next, the above mixture was further combined 1:1 by volume with intestinal fluid and placed in a shaker (110 rpm, 37 °C) for cultivation. After 4 h of cultivation, 100 μL of the appropriate dilution was spread onto TCBS plates to observe whether there is strain growth.

### 2.4. Determination of the Motility and Adhesion Capacity of V. parahaemolyticus

The activated bacteria were diluted to 8 log CFU/mL with TSB medium, mixed 1:1 by volume with gastric acid at pH 4.5, and incubated in a shaker (110 rpm, 37 °C) for 2 h. Then, after centrifugation (12,000 rpm, 2 min), the bacteria were resuspended in phosphate buffered saline (PBS) with OD_600_ adjusted to 0.5–0.6. For controls, activated bacteria were directly centrifuged, resuspended in PBS, and adjusted to the same absorbance. Then, 5 μL of each resuspension was inoculated onto the center of semi-solid medium and incubated at 37 °C. Bacterial motility diameters were measured at 12 h and 24 h. Swimming, swarming, and twitching agar plates were prepared with 1.0, 0.8, and 2 g/dL Luria–Bertani (LB) broth, respectively; 1.5, 0.5, and 1 g/dL agar, respectively; and 2 g/dL NaCl for all plates. The effect of gastric acid on strain adhesion was investigated using crystal violet staining after mixing the above resuspension solution with intestinal fluid in a 1:99 ratio, followed by incubation at 37 °C and 110 rpm [13]. Adhesion was quantified by measuring the absorbance at 600 nm.

### 2.5. Determination of the Survival Rate

Luria–Bertani broth with 1% NaCl and 3% NaCl was prepared. The activated strains were centrifuged, resuspended in PBS, and adjusted to an OD_600_ of 0.5–0.6. Then, 200 μL of resuspended bacterial solution was added to 5 mL of LB medium with different salinities for pre-adaptation until the bacteria grew to the same logarithm. After the culture was completed, the bacterial solution was centrifuged and resuspended with gastric acid at a pH of 4.5. The resuspended mixture was placed in an incubator (37 °C, 110 rpm, 2 h). The appropriate gradient was diluted with PBS every 30 min, and then 100 μL was taken for spreading count.

### 2.6. Preparation and Pretreatment of Litopenaeus vannamei

*Litopenaeus vannamei* (*L. vannamei*) was purchased from a Shanghai fresh food supermarket. The shrimp were shelled, and three groups of samples were prepared, each weighing 4.00 ± 0.5 g. Subsequently, all samples were irradiated with ultraviolet light on an ultra-clean table for 1.5 h [14,15]. Post-irradiation, the samples were homogenized in bags with 12 mL of PBS for 1 min. Finally, 200 μL of the above solution was spread on tryptone soy agar (TSA) plates to check whether the background microorganisms were removed.

### 2.7. V. parahaemolyticus Growth on Shrimp Samples

A 6 mL volume of activated bacterial solution (approx. 7 log CFU/mL) was mixed with 500 mL of 3% NaCl solution to generate a bacterial suspension [16]. Shrimp samples, with background microorganisms removed, were placed in this suspension and shaken for 40 min. After bacterial inoculation, the shrimp samples were placed in an incubator. Samples cultured for 3, 6, 12, 24, 36, and 48 h were selected for microbial enumeration and were homogenized in sterile bags with 12 mL of sterilized saline for 3 min and then gradient-diluted with PBS. Then, 100 μL of the diluted solution was spread on TCBS plates. Another set of shrimp samples stored under the same conditions were homogenized in 27 mL of deionized water for 1 min, and the pH change was measured using a pH meter [17].

### 2.8. Determination of the Survival Rate of V. parahaemolyticus in Different Matrices During Gastric Acid Digestion

*L. vannamei* samples, with background microorganisms removed, were homogenized in homogenizer bags containing 12 mL of 3% NaCl solution (5000 rpm, 8 min). *L. vannamei* homogenate, TSB (3% NaCl), peptone water (PW), and 1 × PBS buffer were prepared as different matrices. The activated bacterial solution was diluted to approximately 6–6.5 log CFU/mL with the respective matrix. The diluent was mixed with the simulated gastric fluid at a ratio of 1:1 (*v*/*v*) and then incubated in a 37 °C shaker (110 rpm, 2 h) [8,18]. The supernatant was diluted appropriately, and 100 μL of the diluent was spread on TSA plates.

### 2.9. The Recovery of V. parahaemolyticus Under SIF

The activated bacterial solution was diluted to approx. 7 log CFU/mL, mixed at a 1:1 ratio with gastric acid, and incubated in a shaker (110 rpm, 37 °C) for 2 h. Then, 5 mL of this mixture was diluted to approx. 3 log CFU/mL, mixed at a 1:1 ratio by volume with intestinal solution, and incubated in a shaker (110 rpm, 37 °C). Samples were taken from the intestinal mixture every 2 h, and the absorbance was measured at 600 nm to monitor bacterial growth. For controls, the activated bacterial solution (3 log CFU/mL) was mixed 1:1 by volume with intestinal solution and similarly incubated and measured.

Based on continuously measured OD_600_ values, the recovery growth curve of *V. parahaemolyticus* in intestinal fluid was plotted over time. The modified Gompertz model was used to simulate the growth of *V. parahaemolyticus* in intestinal fluid both with and without gastric acid treatment. The model was fitted using OriginPro2021 software (Origin Lab Corp., Northampton, MA, USA).

y = A + c exp {−exp[*μ_max_* (λ − t)/A + 1]} (1)

In Equation (1), A represents the minimum additions of strains (CFU/mL), c represents the concentration of bacteria at the highest part of the curve minus A (CFU/mL), *μ_max_* represents the maximum specific growth rate, λ represents the time when the strain started to grow (h), and y represents the number of bacteria at a certain moment (CFU/mL).

### 2.10. Scanning Electron Microscopy (SEM) Assessment of the Morphological Changes in V. parahaemolyticus

The activated bacterial solution was mixed with gastric acid at a 1:1 volume ratio and incubated at 110 rpm and 37 °C for 2 h. After incubation, a portion of the above mixture was prepared for sampling as described below, while the remaining mixture was further incubated with intestinal fluid at a 1:1 volume ratio for either 4 or 8 h at 110 rpm and 37 °C. Similarly, the mixtures after 4 and 8 h of incubation were also prepared for sampling using the same method.

The bacterial solution treated under different conditions was placed in a centrifuge tube and centrifuged for 10 min. After centrifugation, the supernatant was discarded, and the pellet was suspended in PBS to adjust the bacterial concentration to about 8 log CFU/mL. At the end of refrigeration, 1 mL of bacterial liquid was added to a centrifuge tube, and the sample was centrifuged for 2 min. After centrifugation, the supernatant was discarded. Then, 2.5% glutaraldehyde was added for fixation, and the sample was placed in a 4 °C refrigerator for 8 h. Next, the sample was centrifuged again, and the supernatant was discarded. Gradient dehydration was performed with alcohol. The bacterial suspension was suspended with 400 μL of anhydrous ethanol, and 10 μL of bacterial droplets was placed on a circular coverslip. The bacteria were imaged using a Hitachi SU5000 SEM (Hitachi, Tokyo, Japan) at 15.0 kV and 5000× (5.00 K) magnification, with scale bars representing 10.0 microns.

### 2.11. RNA Extraction and Transcriptome Sequencing

RNA was extracted from the VPE28 strain in pure culture, treated with pH 4.5 gastric acid for 2 h, treated with intestinal fluid for 4 h after pure culture, and finally treated first with pH 4.5 gastric acid for 2 h and then treated with intestinal fluid for 4 h. Extraction was performed using the Bacteria RNA Extraction Kit (Majorbio, Shanghai, China). For each treatment group, triplicate samples were prepared, and the bacterial pellets obtained from the treatments were processed at Shanghai Meiji Biotechnology Co., Ltd. (Shanghai, China). The purity and integrity of the extracted RNA were tested using a NanoDrop2000 (Thermo Fisher Scientific, Shanghai, China) and agarose gel electrophoresis. The RNA from the delivered samples was tested to ensure that it was free of impurity contamination and of high quality, making it suitable for use. The extracted RNA was subjected to rRNA removal using the RiboCop rRNA Depletion Kit for Mixed Bacterial Samples (lexogen, Greenland, NH, USA), which indirectly obtained mRNA that had been randomly fragmented into smaller fragments (200–300 nt). This mRNA was subsequently reverse transcribed to synthesize double-stranded cDNA using random primers. The second strand of cDNA was synthesized using dUTP instead of dTTP. The synthesized double-stranded cDNA was modified by adding End Repair Mix to generate blunt ends, phosphorylated at the 5′ end, and an A base was added at the 3′ end. It was then ligated to a Y-shaped sequencing junction. The second strand of cDNA containing dUTP was degraded using the uracil-N-glycosylase enzyme, ensuring that the library exclusively comprised the first strand of cDNA. RNA library construction was performed using Illumina Stranded mRNA Prep, Ligation from Illumina (San Diego, CA, USA). RNA-seq double-ended sequencing was performed using Illumina NovaSeq X Plus (Illumina, San Diego, CA, USA). The read data are available in the NCBI Sequence Read Archive (SRA) under the accession number SRP550378.

### 2.12. Real-Time Quantitative Reverse Transcription PCR (RT-qPCR)

RNA was extracted from the VPE28 strain in pure culture, treated with pH 4.5 gastric acid for 2 h, and treated first with pH 4.5 gastric acid for 2 h and then treated with intestinal fluid for 4 h, by the total RNA extraction kit (TIANGEN, Beijing, China). The total RNA was then converted into cDNA by the Fastking cDNA Kit (TIANGEN). RT-qPCR experiments were performed according to the 2×Realab Green PCR Quick Mix Reagent (LABLEAD, Beijing, China). The selected genes and primer sequences are listed in Table 1. The relative gene expression was calculated using the 2^−ΔΔCt^ method [19].

### 2.13. Statistical Analysis

Statistical analyses were performed by independent sample *t*-tests under a 95% confidence interval through SPSS 17.0., with the significance level set at *p* < 0.05. Images were drawn with Origin 2021. The transcriptome data were processed using the cloud platform (https://analysis.majorbio.com accessed on 6 April 2025) with GCA_000196095.1 as the reference genome. DESeq2 1.40.2 software was used to identify differentially expressed genes (DEGs), and the false discovery rates were controlled by the Benjamini–Hochberg method. The criteria for screening DEGs were a *p*-adjusted < 0.05 and |log2 fold change| (|log2 FC|) ≥ 1. Protein–Protein Interaction was constructed using STRING Networks (https://string-db.org/, accessed on 2 March 2025).

## 3. Results and Discussion

### 3.1. The Experimental Gastric Acid Value

After gastric acid treatment at pH 2.5 for 2 h, all four strains died and could not be resuscitated in the intestinal fluid. As shown in Table 2, strains VPE5 and VPE7 could be resuscitated in intestinal fluid only at pH 4.5 or higher, while strains VPE27 and VPE28 could be resuscitated at pH 3.5 or higher. After 4 h of incubation, four strains treated with pH 4.5 gastric acid showed similar numbers of resuscitated bacteria. Thus, pH 4.5 was chosen for further experiments. Based on previous research, gastric acid at pH 4.5 is typically used as the bacterial treatment condition (37 °C, 110 rpm, 2 h) [8,12,20].

### 3.2. The Motility and Adhesion Capacity of V. parahaemolyticus

The presence of flagella is essential for the infection of intestinal pathogens [21]. *V. parahaemolyticus* has a dual flagellar system that is suitable for locomotion in different situations [22]. Polar flagella are the inherent structure of *V. parahaemolyticus*, and their protein expression is related to swimming ability. The lateral flagella are composed of six different flagellin proteins with sheaths, which contribute to strain attachment and are associated with swarming ability [23,24]. By analyzing the diameter of the rings formed with *V. parahaemolyticus* on different types of agar plates, the effect of gastric acid treatment on the flagella and motility of *V. parahaemolyticus* was investigated.

**Figure 1:**
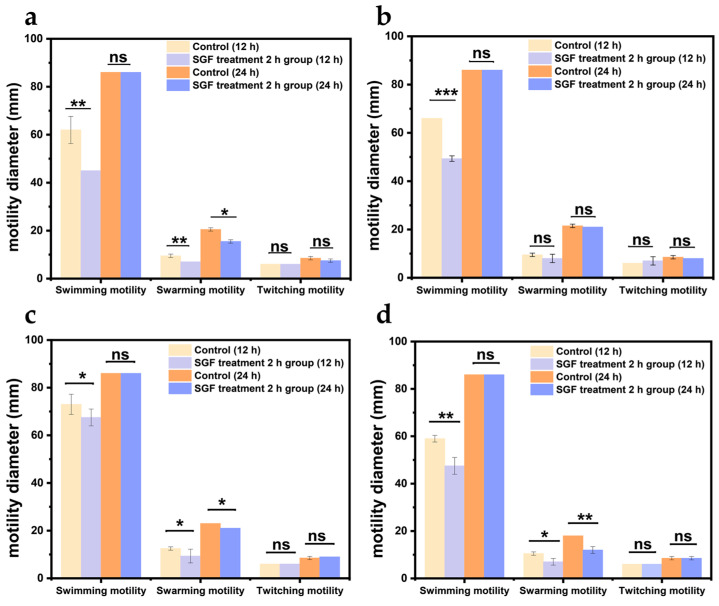
The swimming, swarming, and twitching diameter of *Vibrio parahaemolyticus* treated with simulated gastric fluid (SGF) in which measurements were taken 12 h and 24 h after inoculation. (**a**) VPE5, (**b**) VPE7, (**c**) VPE27, (**d**) VPE28. Data are presented as mean ± SD, *n* = 3. ns > 0.05, * *p* < 0.05, ** *p* < 0.01, *** *p* < 0.001.

In Figure 1, the swimming diameter of the four strains treated with gastric acid was significantly reduced (*p* < 0.05), and the bacterial swarming diameter of strains VPE5, VPE7, and VPE28 decreased from 18–21 mm to 11–15 mm with a significant change (*p* < 0.05). This showed that both polar and lateral flagella of *V. parahaemolyticus* treated with gastric acid were adversely affected.

**Figure 2:**
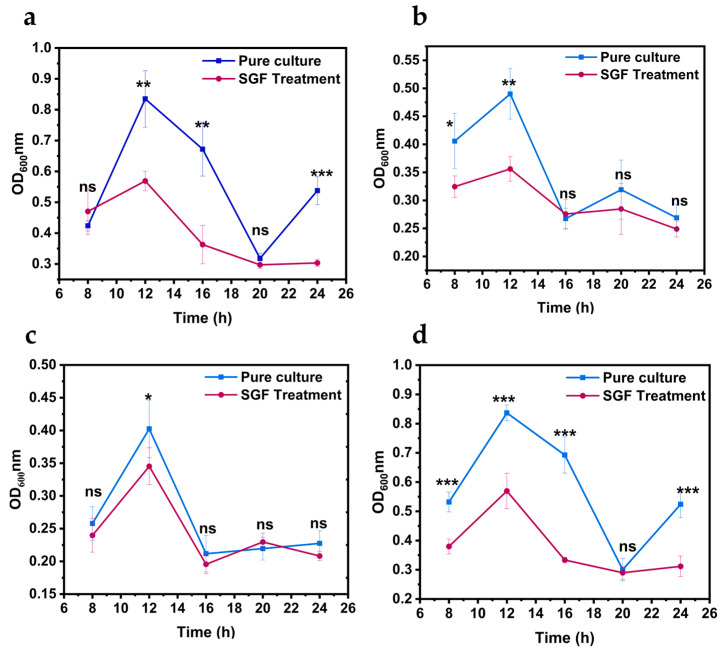
Adhesion capacity of *Vibrio parahaemolyticus* in the intestinal fluid environment before and after SGF treatment. (**a**) VPE5, (**b**) VPE7, (**c**) VPE27, (**d**) VPE28. Data are presented as mean ± SD, *n* = 4. ns > 0.05, * *p* < 0.05, ** *p* < 0.01, *** *p* < 0.001.

Crystalline violet staining experiments revealed that the adhesion ability of the strains decreased after gastric acid treatment, as shown in Figure 2. It was hypothesized that the ability of *V. parahaemolyticus* to colonize the small intestine after passing through the gastric acid barrier may be diminished. The transcriptome sequencing analysis (s0_vs_c0) results support the above experimental results and also explain the mechanism at the molecular level.

### 3.3. Determination of Gastric Acid Resistance of V. parahaemolyticus After Pre-Adaptation with Different Salinities

**Figure 3:**
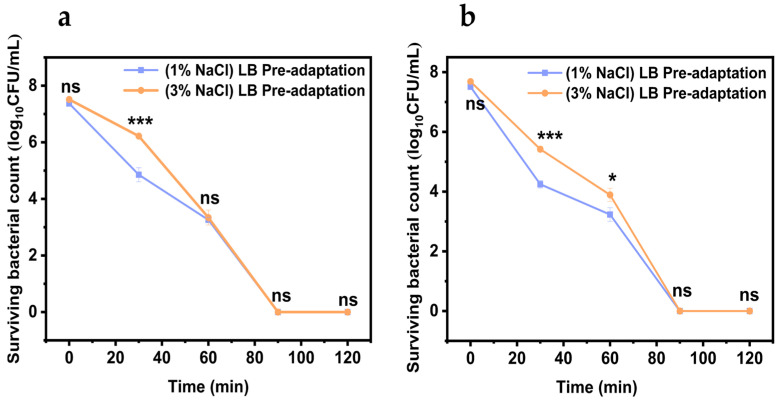
The survival counts of *Vibrio parahaemolyticus* pre-cultured in 1% or 3% NaCl Luria–Bertani (LB) medium upon pH 4.5 gastric acid stress. (**a**) VPE27, (**b**) VPE28. Data are presented as mean ± SD, *n* = 3. ns > 0.05, * *p* < 0.05, *** *p* < 0.001.

As shown in Figure 3b, when the strain was exposed to gastric acid for 30 min, the survival count of strains pre-adapted to high-salt conditions was 10 times higher than that of un-adapted strains. When the strain was exposed to gastric acid for 60 min, the number of strains pre-adapted to high-salt conditions that survived was 10 times higher than that of non-adapted strains. The death rate of *V. parahaemolyticus* decreased after pre-adaptation to high-salt conditions when exposed to gastric acid stress.

It was hypothesized that the higher Na^+^ concentration in the high-salt environment provides energy for the strains [25,26], enabling them to develop a complete systematic flagellar motility system and to adapt more quickly to unfavorable environments. In particular, as shown in Figure 3a,b, the number of strains pre-adapted to high-salt conditions remained at a high level of more than 5 log CFU/mL when the strains were exposed to gastric acid for 30 min. Therefore, pre-adaptation to the high-salt environment improved the ability of *V. parahaemolyticus* to tolerate acid stress.

### 3.4. Growth of Vibrio parahaemolyticus on L. vannamei Samples

*L. vannamei* were chosen as the experimental model. The ability of *V. parahaemolyticus* to grow under fluctuations in food pH was studied, providing data to support a more accurate simulation of the relationship between microorganism growth and the surrounding environment.

**Figure 4:**
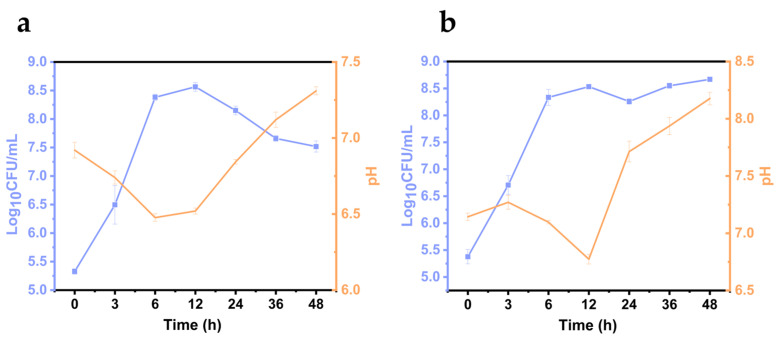
Growth of *Vibrio parahaemolyticus* on *Litopenaeus vannamei* (*L. vannamei*) samples. (**a**) VPE27, (**b**) VPE28. Data are presented as mean ± SD, *n* = 3.

As shown in Figure 4a,b, *V. parahaemolyticus* grew rapidly on *L. vannamei* samples, showing exponential growth within 12 h after inoculation. Over the next 10 h, the number of bacteria remained constant or decreased slightly. After 24 h of inoculation, the bacterial counts remained stable or showed a tendency to decrease, which may be due to the depletion of nutrients and the poor living environment. During *V. parahaemolyticus* growth, the *L. vannamei* pH fluctuates in the range of 6.48–8.18. The significant change in pH (*p* < 0.05) observed during the storage of *L. vannamei* samples did not affect *V. parahaemolyticus* reproduction. When the pH of *L. vannamei* samples decreased to a slightly acidic environment, *V. parahaemolyticus* still remained in a reproductive state, suggesting that *V. parahaemolyticus* has evolved acid adaptation. In Japan, slightly acidic sushi (rice containing vinegar, pH 4.3–4.9) has been repeatedly associated with outbreaks of *V. parahaemolyticus* infections [27]. This highlights the need for enhanced monitoring of slightly acidic foods for potential *V. parahaemolyticus* contamination.

### 3.5. Effect of the Food Matrix on the Escape of V. parahaemolyticus from Gastric Acid Stress

Poisoning incidents caused by *V. parahaemolyticus* are mostly associated with the consumption of uncooked, unboiled, or contaminated foods [14]. *V. parahaemolyticus* may attach to food particles and reach the intestine after ingestion, causing disease [28]. The order of nutrient composition and viscosity of different substrates from highest to lowest is *L. vannamei* homogenate, TSB, PW, and PBS buffer, which were prepared as matrices to simulate the ability of *V. parahaemolyticus* to cross the gastric acid barrier when consuming different foods.

**Figure 5:**
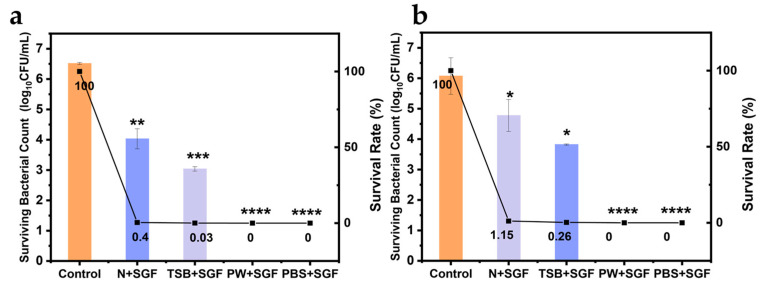
Survival of *Vibrio parahaemolyticus* in four different matrices under gastric acid stress: *L. vannamei* homogenate, trypticase soy broth (TSB), peptone water (PW), and phosphate buffered saline (PBS). (**a**) VPE27, (**b**) VPE28. “N + SGF”, “TSB + SGF”, “PW + SGF”, and “PBS + SGF” represent that *Vibrio parahaemolyticus* was treated with gastric acid for 2 h in a homogenate matrix of *L*. *vannamei,* TSB, PW, and PBS, respectively. Data are presented as mean ± SD, *n* = 3. * *p* < 0.05, ** *p* < 0.01, *** *p* < 0.001, **** *p* < 0.0001.

In Figure 5a,b, the survival rate of *V. parahaemolyticus* was the highest when the shrimp homogenate was digested by gastric acid, and the survival rate of pathogenic VPE28 strain was up to 1.15%. TSB medium is commonly used for the pre-enrichment of *V. parahaemolyticus* [29]. When TSB medium was used as a food matrix under simulated conditions, the survival rate of *V. parahaemolyticus* after exposure to gastric acid was less than 0.5%. PW (containing less nutrients) and PBS buffer (without nutrients) are generally used as cell diluents [30,31]. When *V. parahaemolyticus* entered gastric acid digestion with these two solutions, it was completely killed. Therefore, it was concluded that the survival rate of *V. parahaemolyticus* after gastric acid stress is also influenced by the type of food consumed. As a physical barrier, the food matrix reduces the bacteria’s exposure to gastric acid and helps neutralize some of the acid.

### 3.6. Determination of the Recovery Capacity of V. parahaemolyticus in Simulated Intestinal Fluid

**Figure 6:**
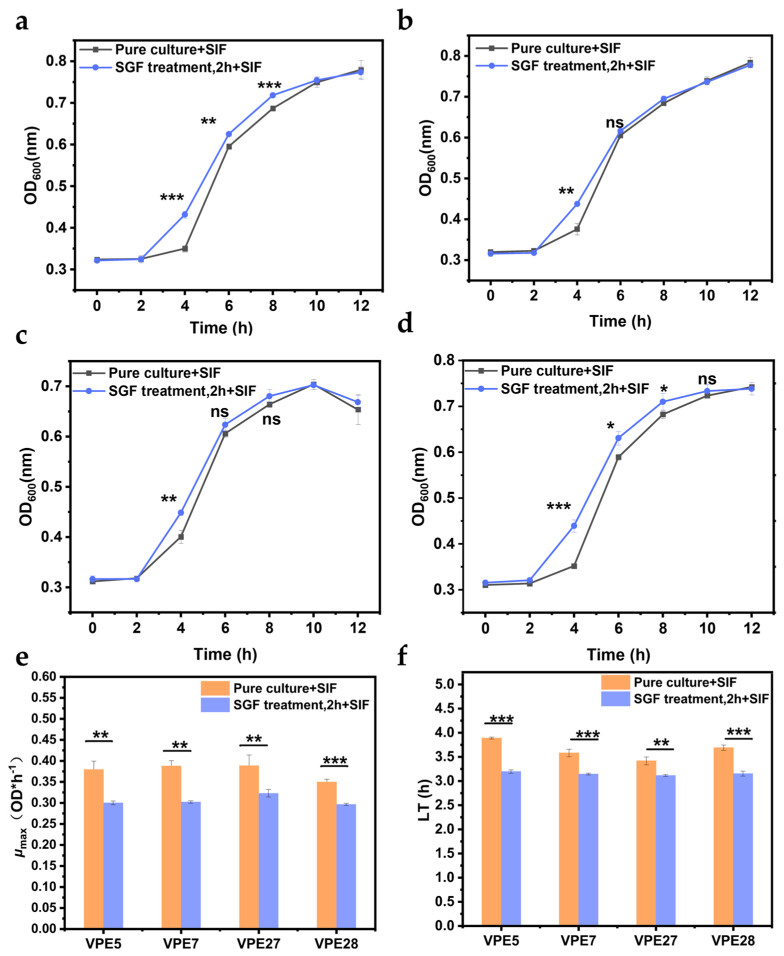
The resuscitation of *Vibrio parahaemolyticus* in simulated intestinal fluid (SIF). (**a**) VPE5, (**b**) VPE7, (**c**) VPE27, (**d**) VPE28. The maximum specific growth rate (**e**) and growth lag phase (**f**) of *Vibrio parahaemolyticus* during resuscitation. Data are presented as mean ± SD, *n* = 3. ns > 0.05, * *p* < 0.05, ** *p* < 0.01, *** *p* < 0.001.

As shown in the recovery curves (Figure 6a–d), *V. parahaemolyticus* that survived gastric acid stress was able to resuscitate in the intestinal fluid. After 4 h of incubation in the intestinal fluid, *V. parahaemolyticus* exposed to gastric acid exhibited a significant growth difference compared with *V. parahaemolyticus* in pure culture; at this point, *V. parahaemolyticus* under the gastric acid treatment showed stronger adaptability. However, this growth difference disappeared after 8 h of incubation in the intestinal fluid, likely due to the nutrient depletion or a decline in bacterial metabolic capacity, leading to the onset of the stabilization period.

Figure 6f shows that the lag phase of *V. parahaemolyticus* treated with gastric acid in the intestinal environment was reduced by about 0.30–0.69 h compared to that of *V. parahaemolyticus* without gastric acid treatment. However, the *μ_max_* (maximum specific growth rate) of *V. parahaemolyticus* treated with gastric acid in intestinal fluid decreased from 0.38 ± 0.04 OD*h^−1^ to about 0.32 ± 0.03 OD*h^−1^, as shown in Figure 6e. This finding indicates that gastric acid treatment does have an adverse effect on *V. parahaemolyticus* growth, potentially due to the death or damage of some bacteria after gastric acid treatment. However, the significant shortening of its lag phase was not expected, indicating that *V. parahaemolyticus* treated with gastric acid could recover more quickly in intestinal fluid, showing stronger adaptability to intestinal fluid.

### 3.7. Changes in V. parahaemolyticus Morphology

**Figure 7:**
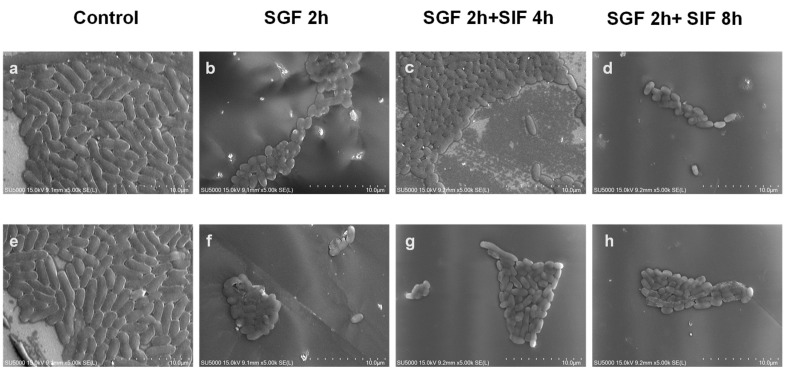
((**a**–**d**): VPE27, (**e**–**h**): VPE28) Scanning electron microscopy images of *Vibrio parahaemolyticus* under different treatment conditions.

Bacteria may alter their morphology when faced with a stressful environment [32]; therefore, the morphology of *V. parahaemolyticus* under gastrointestinal fluid treatment was observed using SEM with the pathogenic strain VPE28 and the non-pathogenic strain VPE27 selected as representative strains. Untreated *V. parahaemolyticus* showed a slender rod shape, and the individuals were independent of each other (Figure 7a,e). As shown in Figure 7b,f, after 2 h of treatment with gastric acid, *V. parahaemolyticus* changed from the original slender rod shape to the spherical shape and adhered to each other. This suggests that some bacteria were damaged and appeared to be in either a sub-lethal or a dead state. In the experimental group where the bacteria were treated with gastric acid for 2 h followed by incubation in intestinal fluid for 4 h, some bacteria were observed to recover from the original spherical shape to an oval or rod shape, and even new slender rod-shaped bacteria were observed (Figure 7c,g). At this point, the bacterial morphology was restored. The results showed that *V. parahaemolyticus* that survived through the gastric acid stress could rapidly recover upon reaching the intestinal environment, allowing it to reproduce to a certain extent and potentially cause disease. However, as time progressed, the depletion of nutrients and the bactericidal effect of bile salts in the intestinal fluid caused the viability of the resuscitated *V. parahaemolyticus* to begin to weaken and to enter a stable period, as shown in Figure 7d,h.

### 3.8. Transcriptomics Analysis

*V. parahaemolyticus* is divided into pathogenic (*trh*^+^ and/or *tdh*^+^) and non-pathogenic (*tlh*^+^/*tdh*^−^/*trh*^−^) strains. Among the pathogenic strains, the detection rate of strains with the genotype *tlh*^+^/*tdh*^+^/*trh*^−^ was relatively high [22,33]. In order to gain a more in-depth understanding of the pathogenicity mechanism of *V. parahaemolyticus* in the human body, a more representative pathogenic strain, VPE28 (*tlh*^+^/*tdh*^+^/*trh*^−^), was selected for transcriptome sequencing. The following four groups were established for transcriptome sequencing: c0 represents pure culture, s0 represents gastric acid treatment for 2 h, c2 represents the pure-cultured bacteria treated with intestinal fluid for 4 h, and s2 represents bacteria treated with gastric acid for 2 h followed by treatment with intestinal fluid for 4 h.

**Figure 8:**
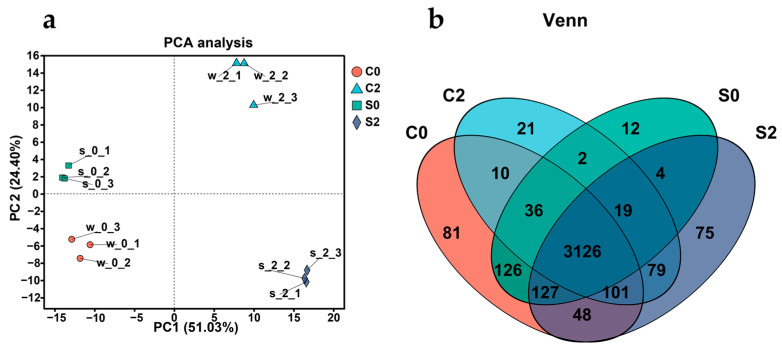
Principal component analysis (PCA) (**a**) and Venn analysis (**b**) between the four treatment groups. Data are presented as mean ± SD, *n* = 3.

In Figure 8a, the four groups of samples showed distinct differences among groups and clustering within groups. The Venn analyses (Figure 8b) revealed a substantial number of differentially expressed genes. Therefore, the quality of the above samples essentially met the experimental requirements.

#### 3.8.1. Exploration of Molecular Mechanisms

**Figure 9:**
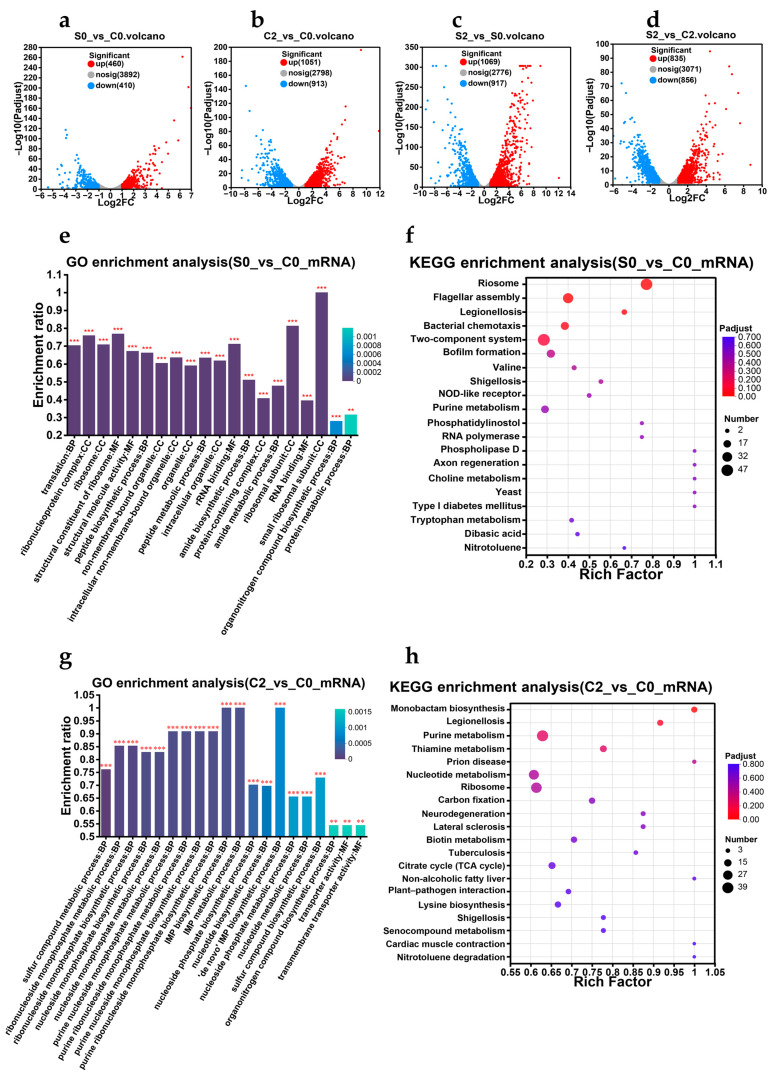

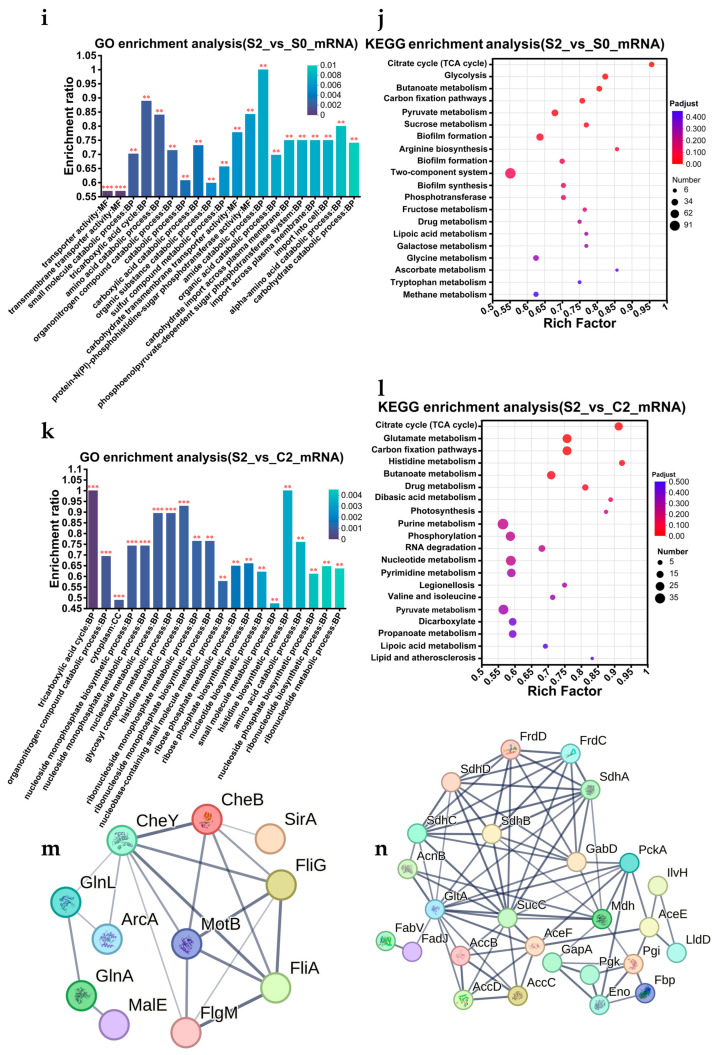
Volcano plots. (**a**) s0_vs_c0 represents s0 as the experimental group and c0 as the control group, (**b**) c2_vs_c0 represents c2 as the experimental group and c0 as the control group, (**c**) s2_vs_s0 represents s2 as the experimental group and s0 as the control group, and (**d**) s2_vs_c2 represents s2 as the experimental group and c2 as the control group. Gene Ontology (GO) annotations and Kyoto Encyclopedia of Genes and Genomes (KEGG) enrichment analyses. (**e,f**) s0_vs_c0, (**g,h**) c2_vs_c0, (**i,j**) s2_vs_s0, and (**k,l**) s2_vs_c2. Protein–protein interaction network analyses. (**m**) Related pathways of *V. parahaemolyticus* passing through the gastric acid barrier, and (**n**) pathways related to the resuscitation of *V. parahaemolyticus*. BP means biological process, MF means molecular function, and CC means cellular component. ** FDR < 0.01, and *** FDR < 0.001 with FDR < 0.05 considered credible data.

Differential gene expression analysis (Figure 9a–d) identified numerous DEGs, which were further analyzed using Gene Ontology (GO) and Kyoto Encyclopedia of Genes and Genomes (KEGG) databases (Figure 9e–l) to identify the top 20 significant pathways.

*V. parahaemolyticus* was very active in resisting gastric acid. In the s0_vs_c0 experimental group (Figure 9e,f), several metabolic pathways were significantly affected (*p* < 0.05), including ribosome biosynthesis, bacterial chemotaxis, flagellar assembly, two-component systems, quorum sensing (QS), ABC transport systems, degradation of amino acids and amines, nitrate respiration, and the catabolism of glucose and fructose. As shown in Figure 9i,j, several pathways are involved during the *V. parahaemolyticus* recovery process, including the membrane transport proteins, transport and metabolism of amino acids, the tricarboxylic acid cycle (TCA cycle), the phosphotransferase system, two-component systems, and ribosomal metabolism pathways.

In summary, *V. parahaemolyticus* not only exhibited an adaptive capacity under gastric acid stress by regulating multiple metabolic pathways but also rapidly adjusted its metabolic strategy during recovery to adapt to the intestinal environment and complete its pathogenic process.

#### 3.8.2. *V. parahaemolyticus* Actively Responds to Gastric Acid Stress Through Various Mechanisms and Prepares for Escaping from Stress at Any Time

QS and two-component systems both rely on sensing and producing the signal molecules to regulate bacterial behavior. QS modulates gene expression by sensing changes in the concentrations of extracellular signaling molecules [34]. As a basic stimulus–response coupling mechanism, the two-component regulatory system completes signal transduction through histidine kinase and response regulator protein, so that organisms perceive and respond to environmental changes [35]. The expression of the sense genes associated with QS, including *che*W, *che*Y, and *mcp*, was significantly upregulated during gastric acid stress; at the same time, the histamine kinase synthesis pathway regulated by the GacS/GacA two-component system was significantly enriched, and the expression of *his*A, *his*IEF, and *his*H and other genes related to histidine metabolism as well as the *tor*R gene, which functions as a DNA-binding response regulator, was significantly upregulated. Previous studies have shown that bacteria could produce a large number of stress proteins when sensing environmental changes. In *Escherichia coli*, the heat shock response can help bacteria cope with the acidic environment [36]. The expression of the regulatory genes *rps*J and *csp*A, which are associated with the stress proteins, was significantly upregulated during gastric acid stress.

Upon sensing this adverse environment stress, *V. parahaemolyticus* conserved its energy while also generating a substantial amount of energy to survive. Specifically, energy-consuming processes such as flagellar assembly and motility were halted, as evidenced by a significant downregulation of genes involved in flagellar assembly, such as *mot*X, *fli*C, and *mot*B. After exposure to gastric acid, the tyrosine metabolism, arginine and tryptophan biosynthesis, and oxidative phosphorylation pathways involved in the TCA cycle of *V. parahaemolyticus* were blocked, resulting in the inability of the bacteria to obtain energy through normal aerobic respiration. Therefore, the bacteria shifted to nitrate respiration, and the upregulation of the *fdo*H, *fdo*I, and *fdo*G genes (with upregulated fold changes of 126.284, 75.572, and 31.832, respectively) led to a significant increase in nitrate reductase expression. The ATP produced by nitrate respiration was used by the ABC transport system, a process in which the bacteria can not only gain energy but can also produce components such as nitrogen and ammonia to neutralize H^+^ in the environment. Subsequently, the ABC transport system acted as a proton pump, and upon receiving signals and energy from nitrate respiration, the *pro*X, *pro*V, *aot*P, and *aap*J genes involved in transport and enzyme synthesis were significantly upregulated, and some protons were rapidly translocated to maintain cell membrane integrity. In addition to maintaining intracellular homeostasis by pumping out protons, the bacteria also rely on the production of ammonia and nitrogen from the breakdown of amino acids and amines to neutralize the intracellular pH levels. Similarly, the upregulation of glycine decarboxylase expression, which produces putrescine to neutralize the acidic substances in the environment during metabolism, was observed. The metabolic pathways of glyoxylic acid and dicarboxylate were significantly enriched. When bacteria use these two pathways for metabolism, they bind to the surrounding H^+^ to maintain cell homeostasis. The significant upregulation of cell membrane synthesis-related genes after gastric acid treatment, such as *dgk*A, *lpx*B, etc., also maintained cell homeostasis

Although rRNA and protein components have highly conserved translation mechanisms, different ribosome subgroups have been reported in bacteria under adverse conditions, such as the S21, L2, and L20 subgroups of *E. coli* under pH 4.5 urea conditions [37,38]. In this study, the ribosomal metabolic pathway was most significantly enriched in *V. parahaemolyticus* (Figure 9e,f), and 27 genes encoding 50S ribosomal large subunit components in *V. parahaemolyticus* were upregulated under SGF treatment (2.48–15.054-fold). Similarly, the expression of 18 components of the small 30S subunit was also upregulated (2.084–13.834-fold). During this process, numerous ribosomes accumulate, allowing *V. parahaemolyticus* to avoid spending excessive time synthesizing new ribosomes once the growth environment becomes suitable, but rather, to immediately upregulate ribosome activity to restore the rapid growth [39]. This could explain why *V. parahaemolyticus* treated with gastric acid can adapt to intestinal fluid (which contains bile salts that kill bacteria) more quickly than bacteria without gastric acid treatment.

#### 3.8.3. *V. parahaemolyticus* That Pass the Gastric Acid Barrier Can Recover and Induce Pathogenicity

As shown in Figure 9j, the two-component system was also involved in the *V. parahaemolyticus* recovery process. As a signal transduction mechanism, the two-component system sensed the environmental change from gastric acid to intestinal fluid. The intestinal fluid has an optimal temperature and a neutral pH, which meet the requirements for *V. parahaemolyticus* recovery. Bacterial resuscitation requires energy. The expression of genes related to the TCA cycle, such as *pck*A, *mdh*, and *frd*C, was significantly upregulated in *V. parahaemolyticus* during the recovery process. The TCA cycle, also known as the citric acid cycle, plays a crucial role in providing energy for metabolism [40]. Intracellular glucose molecules in *V. parahaemolyticus* were broken down into pyruvate, which is subsequently converted to acetyl coenzyme A through pyruvate metabolism. This substance participates as an initial reactant in the TCA cycle, which produces substances such as NADH, FADH2, and ATP [41]. The energy produced was used for nucleotide metabolism and the restoration of the vitality of transmembrane transporter proteins. *V. parahaemolyticus* that has successfully escaped gastric acid stress has accumulated a large number of nucleotides, and most of the energy generated by the TCA cycle at this point was used for the recovery of nucleotide activity, with a small portion used for the generation of new nucleotides.

Transmembrane transporter protein viability is directly associated with bacterial growth, and nutrients in the intestinal fluid will be transported into the cytosol by these proteins when their viability is restored or when they are produced in sufficient quantities [42]. The transcriptomic data indicated that during this process, the *glp*T gene associated with glycerol-3-phosphate transporter synthesis and the *pts*G gene associated with PTS glucose transporter subunit IIBC synthesis were upregulated. The main components of bacterial cell membranes are proteins and phospholipids. The fatty acid synthesis pathway was significantly enriched during resuscitation with the upregulation of the genes *acc*C, VP_RS19205, *fab*V, and *fab*F, which are associated with fatty acid metabolism in *V. parahaemolyticus*. Phospholipids were generated after the conversion of fatty acids to phosphatidic acid by the action of synthesizing enzymes (*glp*K, VP_RS07710, and *glp*D). It was predicted that the repair or synthesis of new cell membranes was occurring in the bacterial cell membrane at this time. Genes regulating flagellar assembly and synthesis, such as *fli*S, *flg*K, *flg*G, and *flg*E, were upregulated in the intestinal fluid, presupposing the restoration of *V. parahaemolyticus* motility. As these processes synchronized, *V. parahaemolyticus* exhibited signs of recovery in the intestinal fluid.

Gastric acid stress appears to be a screening pressure for the survival of *V. parahaemolyticus*. Although the synthesis of phosphoenolpyruvate carboxylase involved in this reaction was increased in both acid-treated and acid-untreated *V. parahaemolyticus* (Figure 9l), the gene *pck*A responsible for the synthesis of this enzyme was significantly upregulated by 9.723–fold in acid-treated *V. parahaemolyticus* compared to the acid-untreated strain. The ability of gastric acid-treated *V. parahaemolyticus* to upregulate the *suc*AB gene of 2-oxoglutarate dehydrogenase involved in the citric acid cycle, the *frd*AB gene of fumaric acid reductase, and the *suc*CD gene of succinyl-CoA synthetase in the intestinal fluid was significantly higher than that of *V. parahaemolyticus* without gastric acid treatment.

As a pathogen, *V. parahaemolyticus* can complete the pathogenic process through the Type III secretion system (T3SS) and VI secretion system (T6SS), adhesins, and hemolytic toxins [43,44]. The unique needle-like structure of T3SS can directly inject virulence proteins into the host cell cytoplasm. T6SS directly injects toxins into target cells upon contact with the host cell [45]. In intestinal fluid, the expression of virulence factors of *V. parahaemolyticus* treated with or without gastric acid treatment in intestinal fluid showed different degrees of upregulation. However, after gastric acid treatment, the virulence genes of *V. parahaemolyticus* changed more significantly. For example, *ysc*F, *ysc*I, and other genes in the Type III secretion system were significantly upregulated, whereas *imp*DEFGH and *imp*M genes in the Type VI secretion system were significantly upregulated. The TssI/VgrG system in the Type VI secretion system was also involved in the expression of virulence proteins. TDH is a cytotoxin that can stimulate intestinal cells to secrete fluid and cause diarrhea [46]. Therefore, the TDH toxin encoded by the *tdh* gene has a direct relationship with the symptoms of food poisoning caused by *V. parahaemolyticus*. Studies have shown that some pathogens have not only evolved resistance to the bactericidal properties of bile but can also use bile as an activator to induce the expression of virulence genes. For example, VtrB, a signal receptor, is induced by bile, which in turn enhances *V. parahaemolyticus* TDH-mediated hemolytic toxicity and T3SS2-mediated enterotoxicity [47,48,49]. The transcriptome data showed that the expression of the *tdh* gene in *V. parahaemolyticus* treated with gastric acid was significantly upregulated (9.413–fold) compared with that of *V. parahaemolyticus* without gastric acid treatment (no significant change). Therefore, *V. parahaemolyticus* treated with gastric acid was more likely to stimulate the intestine and cause diarrhea.

The *V. parahaemolyticus* infection rate varies significantly between populations [22]. People with underlying diseases such as insufficient gastric acid secretion are more susceptible to *V. parahaemolyticus* infection, and it is hypothesized that this may be due to the fact that *V. parahaemolyticus* has a higher survival rate in low levels of gastric acid. After *V. parahaemolyticus* infects the host, the host initiates a series of immune responses, such as the release of pro-inflammatory factors including TNF-α by host cells to reach the site of bacterial infection and the removal of the invading bacteria through autophagic pathways [50,51]. If the host has an immune disease and the system response is delayed, it may cause *V. parahaemolyticus* to proliferate and induce more severe symptoms of infection.

#### 3.8.4. Regulatory Pathway Prediction

Potential interaction targets of DETGs in *V. parahaemolyticus* treated with gastrointestinal fluids were predicted using protein–protein interaction networks. As shown in Figure 9m, there is a possible interaction between the sensing protein CheY and the flagellin FlaM. In Figure 9n, the completion of *V. parahaemolyticus* resuscitation may be centered on the TCA cycle and may mobilize the glycolysis, pyruvate and butyrate metabolism, and prokaryotic carbon fixation pathways to work together.

**Figure 10:**
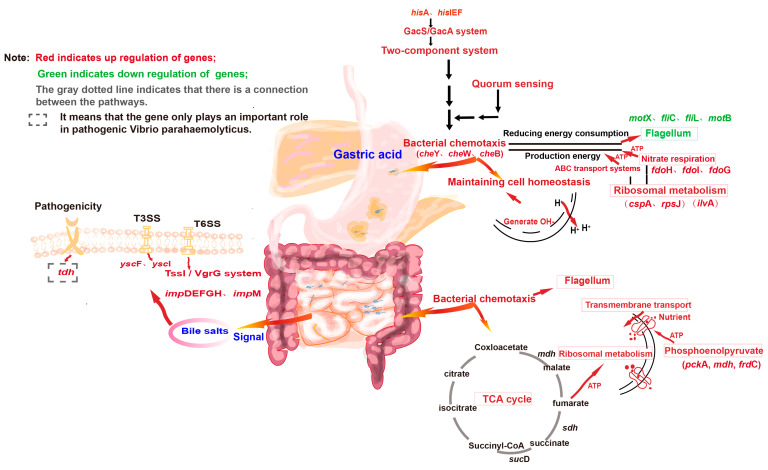
Schematic illustration of *Vibrio parahaemolyticus* escaping the gastric acid barrier, completing resuscitation, and causing disease in humans.

The mechanistic diagram was constructed based on the comprehensive analysis of all transcriptome sequencing data, as shown in Figure 10.

### 3.9. Validation of Transcriptome Data

**Figure 11:**
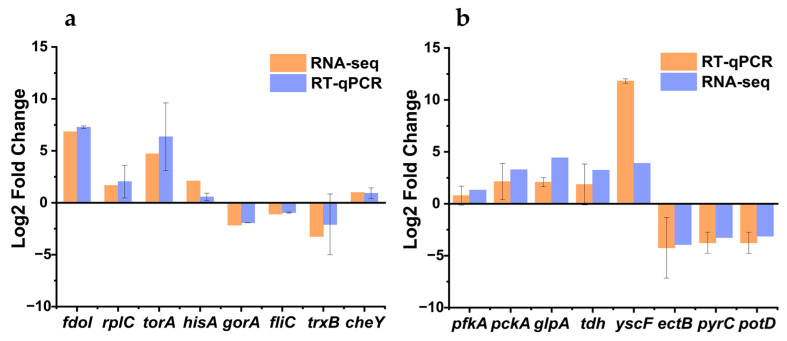
Validation of fourteen genes by RT-qPCR. *Vibrio parahaemolyticus* from the pure culture group, the group treated with gastric acid for 2 h, and the group treated with gastric acid for 2 h and then treated with intestinal fluids for 4 h were used for RT-qPCR experiments. (**a**) s0_vs_c0, (**b**) s2_vs_s0.

The reliability of the transcriptome results of different treatments of *V. parahaemolyticus* was verified by RT-qPCR. The gene expression trend determined by RT-qPCR was consistent with the transcriptome results, as shown in Figure 11. As a result, the transcriptomic results of this study have high credibility.

## 4. Conclusions

Currently, most studies on *V. parahaemolyticus* tolerance and virulence mechanisms have focused on the effects of stress conditions during food processing or storage on the subsequent physiological behavior of *V. parahaemolyticus*. In contrast, less attention has been paid to the effects of stress conditions on *V. parahaemolyticus* tolerance and pathogenicity mechanisms after ingestion through food. In this study, phenotyping experiments performed under static digestion simulation in vitro revealed that *V. parahaemolyticus* was able to pass through the gastric acid barrier and complete recovery using multiple mechanisms. In this process, the acid adaptation exhibited by *V. parahaemolyticus* may provide cross-protection for other stressful conditions, thereby increasing the pathogenicity to humans and increasing the risk of infection. In addition, changes in gene expression before and after gastrointestinal fluid treatment were analyzed using transcriptome sequencing, revealing the survival strategies and pathogenic potential of *V. parahaemolyticus* at the molecular level. By exploring the tolerance mechanisms of bacterial strains to gastric acid and low-salt intestinal environment stresses, this study aims to provide guidance for the more effective control of food-borne pathogenic infections represented by *V. parahaemolyticus*.

## Figures and Tables

**Table 1:** Primers used for real-time quantitative reverse transcription PCR in this study.

Gene	Sequence (5′ to 3′)
16S rRNA-F	TATCCTTGTTTGCCAGCGAG
16S rRNA-R	CTACGACGCACTTTTTGGGA
*fdo*I-F	GCGTTAGAAGGCATGGTGACTG
*fdo*I-R	TGTTTCGCCCATCGCTTCTTTAG
*rpl*C-F	CTGGTACATCTAAGGGTAAAGGTTTCC
*rpl*C-R	GAGCACGGTGAGACAATGAGTTAC
*tor*A-F	TTGTTCCAATCTAAGCCTGACTTCC
*tor*A-R	ATCCACTCCATCTCGCTCATACC
*his*A -F	TCTTCAGGTGGTATCGGCTCATTAG
*his*A-R	TGCCTCCTCTGCTGTGAACTTAC
*che*Y-F	CCTGCTTCGTGACTTGGGTTTC
*che*Y-R	TGGCATATTCCAGTCGGTTACAAC
*gor*A-F	CGCAGGCTACATCGCAGTTG
*gor*A-R	GCTACGCAGTGGTGACTCTTTAC
*trx*B-F	TCGGCGGCGGTAACACAG
*trx*B-R	ATCTTCTCAGCACGGAATGAATCAC
*fli*C-F	GATCATTGATGCGGCACTGAAATAC
*fli*C-R	CGTTCTCGTTAATGTTGTCCAAGTTG
*pfk*A-F	CACTGTGGTGACCTAACTCTGATG
*pfk*A-R	GCGATGCCGTCTTGGATGTTG
*pck*A-F	GGATGACGATGGTGTCTTCAACTTC
*pck*A-R	TGTAGATGTCTGGCTCCGCTTC
*glp*A-F	GCAAGCGATCACACGAGACTAC
*glp*A-R	TGCCACCGAATACTGACAACAATG
*tdh*-F	TTGAAGATGTAATGGCTGAACTAGGC
*tdh*-R	CGACCACCATGATTCATTGTTGTTAG
*vsc*F-F	AACTTCAGCACGCTATCAACAAATG
*vsc*F-R	AGGATCGACTGCATCACATCTTTG
*ect*B-F	CTCTTGCGGACGGTCAGTTG
*ect*B-R	TAAGTGGTGGAAGGAAGCGAATAAC
*pyr*C-F	CCTCGCTCCTATCGTTAATGACTTC
*pyr*C-R	ATGGTAGCGGCAACATTATCAGAC
*pot*D-F	CTACCTAACCGCCGTTACAGAATC
*pot*D-R	CACCAACCGAGTCTTGCCATTC

**Table 2:** The counts of *Vibrio parahaemolyticus* resuscitated in intestinal fluid (resuscitation time: 4 h) after gastric acid treatments at pH 2.5, pH 3.5, and pH 4.5.

pH of the Gastric Acid	The Bacterial Count (log_10_ CFU/mL)			
	VPE5	VPE7	VPE27	VPE28
pH 2.5	0	0	0	0
pH 3.5	0	0	6.05 ± 0.08	7.71 ± 0.08
pH 4.5	7.86 ± 0.02	8.22 ± 0.04	7.76 ± 0.20	8.03 ± 0.09

Data are presented as mean ± standard deviation (SD), *n* = 3 (biological replicates).

## Data Availability

The original contributions presented in this study are included in the article. Further inquiries can be directed to the corresponding author.
